# Comparison of fitness traits and their plasticity on multiple plants for *Sitobion avenae* infected and cured of a secondary endosymbiont

**DOI:** 10.1038/srep23177

**Published:** 2016-03-16

**Authors:** Da Wang, Xiaoqin Shi, Peng Dai, Deguang Liu, Xinjia Dai, Zheming Shang, Zhaohong Ge, Xiuxiang Meng

**Affiliations:** 1 State Key Laboratory of Crop Stress Biology for Arid Areas (Northwest A&F University), Yangling, Shaanxi Province 712100, China; 2 College of Plant Protection, Northwest A&F University, Yangling, Shaanxi Province 712100, China; 3 Department of Foreign Languages, Northwest A&F University, Yangling, Shaanxi Province 712100, China; 4 School of Environment and Natural Resources, Renmin University of China, Beijing 100872, China

## Abstract

*Regiella insecticola* has been found to enhance the performance of host aphids on certain plants, but its functional role in adaptation of host aphids to plants is still controversial. Here we evaluate the impacts of *R. insecticola* infections on vital life-history traits of *Sitobion avenae* (Fabricius), and their underlying genetic variation and phenotypic plasticity on three plants. It was shown that effects of *R. insecticola* on *S. avenae*’s fitness (i.e., developmental time and fecundity) were neutral on oat or wheat, but negative on rye. Infections of *R. insecticola* modified genetic variation that underlies *S. avenae*’s life-history traits. This was demonstrated by comparing life-history trait heritabilities between aphid lines with and without *R. insecticola*. Moreover, there were enhanced negative genetic correlations between developmental time and fecundity for *R. insecticola* infected lines, and structural differences in G-matrices of life-history traits for the two types of aphid lines. In *R. insecticola*-infected aphid lines, there were increases in plasticities for developmental times of first and second instar nymphs and for fecundity, showing novel functional roles of bacterial symbionts in plant-insect interactions. The identified effects of *R. insecticola* infections could have significant implications for the ecology and evolution of its host populations in natural conditions.

Many free-living organisms can harbor fungal, bacterial, or viral endosymbionts that are extremely diverse and encode the vast majority of genes in the biosphere^1^. Indeed, over half of all insect species (the majority of all organisms in terrestrial ecosystems) are estimated to be able to establish endosymbiotic associations with various microorganisms, among which the best studied are those within aphids^2^,^3^,^4^,^5^. For example, most aphids harbor the primary symbiont, *Buchnera aphidicola*, a bacterium that provides some essential amino acids absent in plant phloem sap to its aphid hosts, and is required for its hosts’ survival^6^,^7^. Secondary or facultative symbionts can also be found in many aphids, and they can be vertically transmitted during both sexual and asexual reproduction of their host aphids; occasionally horizontal transfer between host species may also occur^6^,^8^,^9^. Three of the most studied secondary symbionts are *Regiella insecticola* (also known as PAUS or U-type), *Hamiltonella defensa*, and *Serratia symbiotica*, which are γ-Proteobacteria that undergo high rates of vertical transmission. Thus, their fitness hinges upon the survival and reproduction of their host aphids^4^. Indeed, it is well known that the infection of secondary symbionts can confer novel ecological traits to the aphid hosts, such as defense against fungal pathogens, increased parasitoid resistance, and increased thermal tolerance^10^,^11^,^12^,^13^,^14^. Impacts of such symbionts on host phenotypes are often thought to be large and relevant to host fitness^3^,^15^. However, it has been found that secondary symbionts’ manipulations through hemolymph injection or eradication with antibiotics have neutral or negative effects on host aphids in some cases^4^,^16^. Therefore, it is still controversial in what conditions or respects facultative symbionts can benefit their aphid hosts^6^.

Evidence is accumulating that secondary symbionts may have significant impacts on the performance of aphids on different host plants. For example, the fecundity of *R. insecticola*-infected strains of the pea aphid, *Acyrthosiphon pisum* Harris, was nearly 50% higher than that of corresponding uninfected strains on white clover, *Trifolium repens* L., suggesting that infections of *R. insecticola* had the potential to enhance the fitness of its host^12^. Several studies have revealed a striking pattern that pea aphid (*A. pisum*) individuals collected from certain plants (i.e., *Trifolium* sp.) worldwide tend to be infected with *R. insecticola*^6^,^17^,^18^,^19^. In addition, host plant ranges and host acceptance behaviors of aphids have also been shown to be linked to the infection of *R. insecticola*^6^,^20^, indicating the potential of this endosymbiont to influence host aphids’ adaptation to certain plants.

Because they represent novel functioning genomes that can be incorporated by their hosts, another benefit secondary endosymbionts may confer to their host aphids is increased genetic variation underlying life-history traits, which may enhance the potential of adaptive changes in these traits of aphids on different plants^3^,^16^,^21^. Phenotypic plasticity (another powerful means of adaptation other than genetic change) can also be important for aphids’ successful use of various host plants, which often occur in spatially and temporally discrete patches and act as differential selective environments^22^,^23^. Thus, we assume that *R. insecticola* can significantly affect its host aphids’ particular life-history traits on different plants, as well as the underlying genetic variation and phenotypic plasticity of these traits. However, studies have been rare in these respects. The English grain aphid, *Sitobion avenae* (Fabricius), a widespread pest on cereals such as wheat, oat and rye around the world^24^,^25^, is a good model to study such effects, because this aphid can be reared clonally in the laboratory, and it was found to be infected with *R. insecticola* naturally^26^. Therefore, clones of *S. avenae* were collected from two provinces of China, and *R. insecticola* infections were detected and manipulated to determine the performance of the host aphid under common laboratory conditions. Specifically, the objectives of this study are to: 1) determine if *R. insecticola* can modify *S. avenae*’s fitness on three host plants; 2) explore the test endosymbiont-mediated genetic variation and phenotypic plasticity among *S. avenae* lines; 3) examine the implications of *R. insecticola* infections for *S. avenae’s* ecology and evolution.

## Results

### Comparison of fitness traits

**Figure 1:**
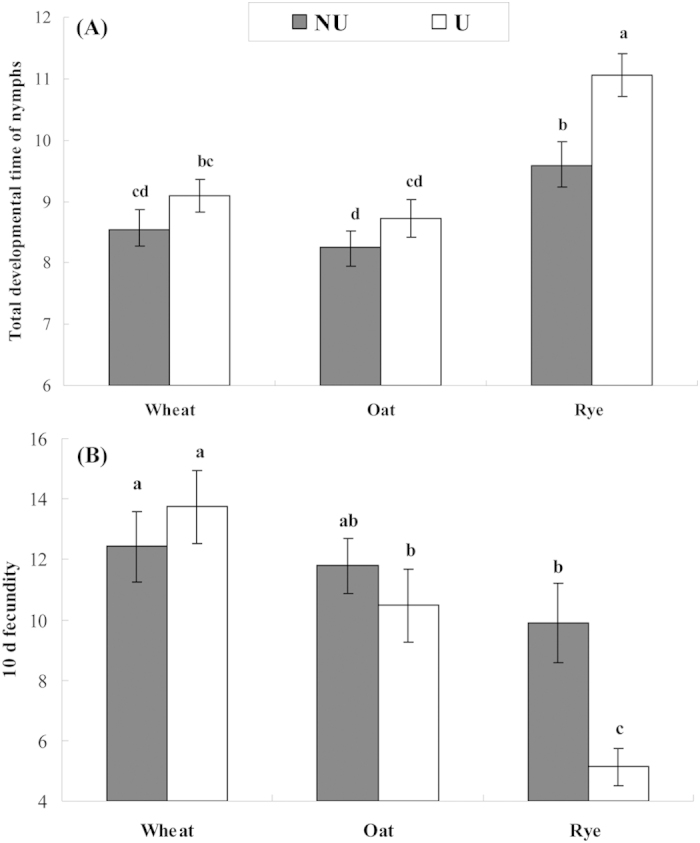
Comparisons of the total developmental time of nymphs (**A**) and 10 d fecundity (**B**) for *Regiella insecticola* – infected (U) and uninfected (NU) lines of *Sitobion avenae* on three host plants (different letters above bars of a particular trait indicate significant differences among treatments at *α* = 0.05).

Seven *R. insecticola* infected lines of *S. avenae* were cured of the endosymbiont successfully. The life-history and fitness traits [i.e., the developmental time of 1^st^ to 4^th^ instar nymphs (DT1-DT4), the total developmental time of nymphs (DT5), and 10 d fecundity] of these cured aphid lines were compared to those of corresponding infected lines. The genetic variation and phenotypic plasticity of these fitness traits were analyzed. Treatment (i.e., the manipulation of *R. insecticola* infection status) altered DT5, although it contributed relatively little (2.9%) to the total variance of the test trait (Table 1). Variance from ‘test plant’ accounted for 10.3% and 7.7% of the total for DT5 and 10 d fecundity, respectively. Interactions of ‘treatment’ and ‘test plant’ were identified for 10 d fecundity, but not for DT5. ‘Clone’ (i.e., clonal lines) nested in ‘treatment’ explained a relatively large proportion of the total variance (i.e., 32.5% and 48.0% for DT5 and 10 d fecundity, respectively). *Regiella insecticola* infected lines showed a longer DT5 than corresponding cured lines on rye, but not on wheat or oat (Fig. 1). Infection of *R. insecticola* showed no positive or negative effects for 10 d fecundity on wheat or oat, but it reduced 10 d fecundity on rye.

### Comparison of genetically-based variation

When tested on wheat, *S. avenae* lines cured of *R. insecticola* presented significant broad-sense heritability only for DT3 among all test life-history traits, whereas the corresponding *S. avenae* lines carrying the endosymbiont showed significant heritabilities for all test traits but DT2 and DT3 (Table 2). On oat, significant heritabilities were found for DT1, DT5 and 10 d fecundity of *S. avenae* lines carrying *R. insecticola*, however, the only significant heritability was found for DT5 of corresponding *S. avenae* lines cured of the test endosymbiont. *Sitobion avenae* lines cured of *R. insecticola* presented significant heritabilities for DT3 and 10 d fecundity, whereas none of the test traits showed significant heritabilities for corresponding *S. avenae* lines carrying *R. insecticola*.

For *S. avenae* lines cured of the test endosymbiont, significant genetic correlations were found between DT5 and all other tested life-history traits; other than DT5, DT4 was also found to be negatively correlated to 10 d fecundity (Table 3). Similar patterns were found for *S. avenae* lines naturally infected with *R. insecticola*. However, negative correlations between DT5 (or DT4) and 10 d fecundity were stronger for *S. avenae* lines infected with *R. insecticola* compared to the corresponding cured aphid lines.

G-matrices for *S. avenae* lines infected and cured of *R. insecticola* were compared using the Flury’s method and jump-up approach (meaning the G matrix structural differences are tested against the hypothesis of unrelated structure at each step in the hierarchy) (Table 4). When tested on wheat, the CPC(3) model best explained the differences between matrices for *S. avenae* lines infected and cured of *R. insecticola* (LRT = 322.3, *P* < 0.001), whereas the matrices for both kinds of aphid lines shared only one principal component [i.e., CPC(1)] on oat (LRT = 41.1, *P* < 0.001). When tested on rye, the differences between G matrices for *S. avenae* lines infected and cured of the test endosymbiont were best explained by the full CPC model (i.e., all principal components shared in common), but the matrices were not equal (LRT = 34.2, *P* < 0.05).

### Fitness trait plasticity and selection of three alternative plants

**Figure 2:**
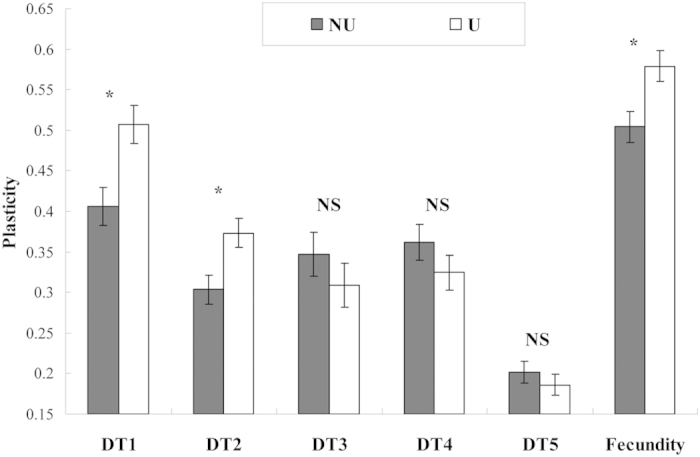
Comparisons of life-history trait plasticities between *Sitobion avenae* lines infected (U) and uninfected (NU) with the test endosymbiont (DT1-DT4, developmental time of 1^st^ to 4^th^ instar nymphs; DT5, total developmental time of nymphs; * and NS, significant and non-significant differences between U and NU at *α* = 0.05 respectively).

The plasticity of DT1 for *S. avenae* lines carrying *R. insecticola* was higher than that for corresponding cured lines (Fig. 2). Increase in plasticity was also found for DT2 and 10 d fecundity of *R. insecticola* infected aphid lines. However, no significant differences between both types of *S. avenae* lines were found for plasticities of DT3, DT4 or DT5.

Selective effects (i.e., selection differentials and gradients) of alternative host plants (i.e., wheat, oat and rye) on life-history trait plasticities for *S. avenae* lines infected and cured of *R. insecticola* were evaluated (Table 5). In response to the three alternative host plants (i.e., alternative environments), *S. avenae* lines cured of the test endosymbiont presented significantly negative differentials for plasticities of all tested life-history traits but DT4, and the directional selection gradient for plasticity of 10 d fecundity of these lines was found to be the only one that was both significant and negative. For *S. avenae* lines naturally infected with *R. insecticola*, significantly negative differentials for plasticities of all test traits were found but those of DT3 and DT4, and significantly negative gradients were found for plasticities of DT1, DT2 and 10 d fecundity.

## Discussion

Earlier studies suggested that *R. insecticola* could have potential positive effects on host aphids’ fitness and adaptation to plants^12^. In this study, the infection of *R. insecticola* showed no significant fitness benefits for its aphid host (*S. avenae*) on wheat and oat in terms of developmental time and fecundity, and it even slightly reduced the fitness of *S. avenae* on rye (increased DT5, and decreased fecundity). Such results indicated that this secondary endosymbiont could have little or no impacts in facilitating utilization of particular plants for its aphid host, and this agrees with the findings of^27^. The differential responses of *R. insecticola* infected aphid clones on three plants also suggested plant-dependent impacts of symbiotic micro-organisms on the fitness of host insects, and this was further substantiated by significant interactions between ‘treatment’ and ‘test plant’ for fecundity in the ANOVA. The plant-dependent fitness effects of *R. insecticola* infections identified in this study were in consistent with the findings of^28^,^29^. Reduced fitness of *S. avenae* lines infected with *R. insecticola* on rye suggested a probable cost of carrying this endosymbiont. This was not unexpected, because the primary functional role of this endosymbiont could be defense against fungal pathogens^15^,^30^,^31^.

It was reported that 34% of collected *S. avenae* clones on multiple host plants [i.e., wheat, oat and cocksfoot grass (*Dactylis glomerata*)] were infected with *R. insecticola* in England^32^. In our study, *R. insecticola* infection levels (ca. 15%) (unpublished data) on wheat were about half of what was reported in the abovementioned study. Nevertheless, the results are consistent with the finding that the frequency of facultative symbionts was often found to range from low to intermediate^5^,^11^,^33^. Because *R. insecticola* infected lines tend to have longer developmental times, and negative genetic correlations between developmental time and fecundity are identified in our study, it’s expected that aphid genotypes that do not harbour this symbiont should be positively selected, decreasing the frequency of this symbiont in the field. The low frequencies of *R. insecticola* occurrences in natural *S. avenae* populations are also in agreement with the neutral and negative effects of this endosymbiont on *S. avenae*’s fitness in our study. Other than China and England, this endosymbiont has been reported from *S. avenae* collected in Germany^32^,^34^, indicating its occurrence and maintenance in broad geographic areas.

Obviously, the maintenance of *R. insecticola* in *S. avenae* populations can not be explained by modified developmental time and fecundity in infected host individuals. However, *R. insecticola* may significantly influence other aspects of the life-history of its host aphids. For example, this endosymbiont was shown to affect the frequency of winged morph production in the pea aphid (*A. pisum*)^35^. In our study, compared to corresponding cured lines, the broad-sense heritabilities of *R. insecticola* infected lines were increased on wheat and oat, but decreased on rye for particular traits (e.g., fecundity), indicating that genetic variation among *S. avenae* populations could be modified by the infection of this endosymbiont. This makes sense since transovarially transmitted microbial symbionts constitute novel functioning genomes incorporated by their host organisms^3^. The negative genetic correlations between fecundity and the total developmental time of nymphs were apparently enhanced for *R. insecticola* infected lines of *S. avenae*. Phenotypic plasticities of vital life-history traits were also shown to be modified by infections of this endosymbiont (e.g., plasticities of DT1, DT2 and fecundity for *S. avenae* lines carrying *R. insecticola* were significantly higher than those for cured lines). The alternative test plants were shown to have relatively stronger direct selections on life-history trait plasticities (e.g., DT1, DT2 and fecundity) of infected lines compared to those of cured lines. One possible explanation is that secondary symbionts could modify plant physiology^3^,^36^, thus creating differential selective environments for the aphid hosts. Collectively, these data suggest that *R. insecticola* infections should play significant roles in the evolutionary dynamics of vital life-history traits in natural *S. avenae* populations. Indeed, significant differences between G-matrices of life-history traits for *S. avenae* lines infected and cured of the test endosymbiont were found on all the three test plants, showing that the G-matrix structure could have a close relationship to the infection status of the endosymbiont. Further studies are needed to determine the impacts of secondary endosymbionts on the stability of G-matrix of life-history traits for the host aphid over time and explore the evolutionary implications.

Overall, our data provide additional evidence that facultative endosymbiotic bacteria can influence many aspects of their arthropod hosts’ life-history. While increased genetic variation among aphid populations resulting from the infection of secondary symbionts may have the potential to facilitate the evolution of adaptive life-history traits in aphids^37^, the increased plasticity of life-history traits (e.g., DT1, DT2 and fecundity) for *S. avenae* lines infected with *R. insecticola* seemed to be maladaptive under common laboratory conditions (shown by significantly negative selection gradients). Therefore, it is likely that there occur tradeoffs between functional roles of endosymbionts for their hosts, although there has been no clear evidence for this^5^. Our study confirms the notion that endosymbiosis (as a complex and dynamic process, instead of an evolutionary end) can have a profound influence on the phenotypic complexity and evolution of herbivorous insects^38^. In order to comprehend fully the evolutionary and ecological processes shaping insect populations (esp., aphid populations), it is thus important to explore the roles of symbiotic bacteria beyond the well-characterized characters (e.g., fungal pathogen and parasitoid resistance^13^,^14^,^39^, and thermal tolerance^40^), and to develop new models for the dynamics of endosymbiont-mediated coevolution that incorporate changing genetic variation, modified phenotypic plasticity, and tradeoffs between symbionts’ functional roles.

## Methods

### Aphid colony

Aphid samples were collected from wheat (cultivar unknown) fields in the provinces of Qinghai and Shaanxi in China from April to July in 2013 (Table 6). Colonies of collected clonal lines were established by caging these parthenogenetic aphids individually on wheat (*Triticum aestivum* L. cv. ‘Aikang 58’) seedlings in the lab as detailed previously in^22^. Collected clones were genotyped at four microsatellite loci (Sm10, Sm12, Sm17, and S4aΣ) as described previously^37^ (Table 6, also see^41^ for more details). By this approach, the seven *S. avenae* clones used in this study were confirmed as being genetically distinct from one another. All collected *S. avenae* clones were reared on ‘Aikang 58’ for at least three generations prior to this study, in order to minimize confounding effects from prior experience of different host plants.

### Detection of *Regiella insecticola*

Whole-insect DNA extractions were conducted as described in^42^. The bacterial 16S rDNA was amplified with universal primers 16SA1 (5′-AGAGTTTGATCMTGGC TCAG-3′) and 16SB1 (5′-TACGGYTACCTTGTTACGACTT-3′) following^43^. PCR reactions were run on 2% agarose gels, and the products were then cloned and sequenced. Cloning of PCR products was carried out as described in^42^. Sequences of cloned 16S rDNA fragments were obtained using the sequencing facility at Sangon Biotech (Shanghai, China). The resulting sequences were blasted, and they were found to be up to 98% identical with previously published sequences of *R. insecticola* (e.g., AY296734^44^), from the pea aphid (*A. pisum*). *Hamiltonella defensa*, *Ricketssia* sp., and the obligate symbiont, *Buchnera aphidicola*, were also found in our collected *S. avenae* samples (data not shown). The resulting sequences and previously reported sequences in GenBank were used to construct diagnostic primers (forward: 5′-AGAGTAATATGCTTATCGATTG-3′; reverse: 5′-GCTCGCCGCTCTTTGTAT-3′). Diagnostic PCR analysis was conducted by using the following temperature program: 94 °C for 5 min, followed by 35 cycles consisting of 94 °C for 0.5 min, 55 °C for 1 min, and 72 °C for 1.5 min. Appropriate negative and positive controls were used when conducting PCR reactions. Seven *S. avenae* clones (collected on wheat, *Triticum aestivum* L.) were found to harbor *R. insecticola*, which was eradicated by antibiotic treatments (see below) to establish corresponding uninfected clonal lines within the same genetic background. Both sequencing and diagnostic PCRs were used to determine and confirm the infection status of *R. insecticola* in each aphid line. The presence or absence of *R. insecticola* was also assessed on siblings of the aphid individuals whose life-history traits were characterized. The *R. insecticola* 16S rDNA sequences from all infected clones were the same (Genbank accession number, KT428726).

### Curing of *R. insecicola* infected lines

Natural *R. insecticola* infections were cured for the above-mentioned seven aphid clones through oral administration of antibiotics. Cut wheat stems were placed in 1.5 ml Eppendorf tubes containing 100 μg ml^−1^ ampicillin, 50 μg ml^−1^ cefotaxime and 50 μg ml^−1^ gentomicin, and second instar nymphs of *S. avenae* were allowed to feed on them for 4–6 days at 20 °C^27^,^45^. Wheat seedlings at the one-two leaf stage were used to rear the surviving aphids. Second generation aphid nymphs were checked for the presence or absence of *R. insecticola* using specific primers mentioned above. Offspring of the adults found uninfected with *R. insecticola* were used to establish clonal lines. The cured lines were tested for the lack of *R. insecticola* for at least six generations after antibiotic treatments^27^. A total of seven cured lines were founded using this approach. The *R. insecticola*-infected and cured aphid lines were maintained on wheat seedlings (cv. Aikang 58), and were regularly checked for *R. insecticola* infection by diagnostic PCR. The infection status of *R. insecticola* was also reconfirmed following life-history bioassays.

### Life-history bioassays

The seven *S. avenae* clonal lines carrying *R. insecticola* and the seven corresponding cured lines were then used in life-history bioassays, which were conducted as described previously^22^,^46^. Briefly, wingless adults of the seven naturally infected *S. avenae* lines and their corresponding cured lines were transferred to fresh wheat seedlings at the one- to two-leaf stage (one adult per plant), which were enclosed individually in a transparent container (having a Terylene mesh top for ventilation; 6 cm in diameter and 15 cm in height). Each pot of seedlings was checked two to three hours later to ensure that all aphid individuals but one newborn nymph were removed from each plant. Wheat seedlings with test newborn nymphs were placed in growth chambers with the following conditions: a temperature of 20 ± 1 °C, a relative humidity of 65 ± 2%, and a photoperiod of L16 : D8. Five to ten replicates were conducted for each *S. avenae* line. We monitored the developmental status, number of nymphs produced (newborn nymphs were counted and then removed), and death events of test aphid individuals daily until day 10 after reproduction was initiated for each test individual. Test plants were replaced with fresh ones weekly. Winged and wingless aphids have differential performance in terms of fecundity, so we only considered replicates of wingless aphids (the most common morph in all tests) in the following analysis^32^.

### Statistical analyses

As described previously in ref. 24, the developmental times of nymphs (DT1-DT5), and 10 d fecundity were calculated. These traits were analyzed by using three-way nested analyses of variance (ANOVA) in SAS^47^. Effects of ‘treatment’ (i.e., elimination of *R. insecticola* using antibiotics), ‘test plant’ (i.e., wheat, oat and rye), ‘clone’ nested in ‘treatment’, and the interactions between the first two factors were analyzed following Dai *et al*.^48^. Treatment means were separated using Tukey tests at α = 0.05 following significant ANOVA. When necessary, log-transformation of data was conducted to meet the requirements of normality and homoscedasticity for these analyses.

Clonal genotypes were used in our aphid life-history bioassays, and such experimental designs allowed us to partition the total variance of a particular life-history trait (*V*_*P*_) into within-clone components *V*_*E*_ (i.e., environmental variance or residual variance) and among-clone genetic components *V*_***G***_ (i.e., the broad-sense genetic variance)^25^. The restricted maximum likelihood (REML) method was applied to evaluate phenotypic variances, and genetic variances and covariances for life-history traits with the software VCE 6.0.2 49. As detailed previously in ref. 22, we then estimated broad-sense heritabilities of traits as 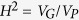 (1), and genetic correlations between traits *x* and *y* as 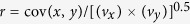
(2) (cov[*x, y*], the genetic covariance between *x* and *y* ; *v*_*x*_ and *v*_*y*_, genetic variance of *x* and *y*). Paired G matrices were compared with the Flury hierarchical method in the software CPCrand^50^, which could determine structural differences between G matrices by assessing their eigenvectors and eigenvalues as described in ref. 50. G-matrix structural models of unrelated structure, partial common principal components, common principal components, proportionality, and equality can be tested in order with this software (see also in ref. 51. Likelihood-ratio tests (LRTs) were used to identify the statistical significance of genetic correlations and broad-sense heritabilities following^51^.

As described previously in ref. 22, the amount of plasticity for the abovementioned life-history traits of *S. avenae* clonal lines on the three alternative host plants (i.e., wheat, oat and rye) was assessed by determining the coefficient of variation with the equation 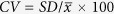
(3), where *SD* is the standard deviation of each treatment, and 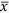 is the mean of each treatment. The strength of selection of alternative host plants on life-history trait plasticity of *S. avenae* lines was evaluated through determining selection differentials and gradients with the PROC REG procedure in SAS following^22^. Briefly, the relative fitness of each aphid line was assessed by dividing the clone’s 10 d fecundity by the mean of all test lines under each treatment, and all trait plasticity data were standardized to have mean zero and unit variance. Simple linear regressions were used to evaluate standardized selection differentials (i.e., the total strength of selection on each character including direct selection and indirect selection from correlation with other characters), and multiple regressions were performed to determine standard linear selection gradients (i.e., the strength of direct selection excluding the effects of indirect selection) [see more details in refs 52,53.

## Additional Information

**How to cite this article**: Wang, D. *et al*. Comparison of fitness traits and their plasticity on multiple plants for *Sitobion avenae* infected and cured of a secondary endosymbiont. *Sci. Rep*. **6**, 23177; doi: 10.1038/srep23177 (2016).

## Figures and Tables

**Table 1:** Estimates of variance components for life history traits of *Sitobion avenae* clones showing main effects of treatment (i.e., antibiotic removal of *Regiella insecticola* in aphid clones), test plant (plant), clone nested in treatment and interactions (significant effects highlighted in boldface type).

Traits	Variance source	df	*F*	*P*	% total
Total developmental time of nymphs	Treatment	1	13.15	**<0.001**	**2.9**
	Plant	2	23.67	**<0.001**	**10.3**
	Treatment × plant	2	1.81	0.211	0.8
	Clone (treatment)	12	12.46	**<0.001**	**32.5**
	Error	246	**–**	**–**	53.5
10 d fecundity	Treatment	1	1.5	0.294	0.3
	Plant	2	22.63	**<0.001**	**7.7**
	Treatment × plant	2	6.09	**0.012**	**2.1**
	Clone (treatment)	12	23.49	**<0.001**	**48.0**
	Error	246	**–**	**–**	41.9

**Table 2:** Broad-sense heritabilities of life history traits for *Sitobion avenae* lines infected (U) and uninfected (NU) with *Regiella insecticola* on three test plants [table entries are mean (SE); DT1-DT4, developmental time of 1^st^ to 4^th^ instar nymphs; DT5, total developmental time of nymphs; statistical significance (*P < 0.05; **P < 0.01; ***P < 0.001) of heritability for a trait evaluated using likelihood-ratio tests].

Traits	Wheat		Oat		Rye	
	U	NU	U	NU	U	NU
DT1	0.4387*	0.2132	0.4485*	0.1941	0.2327	0.2844
	(0.1162)	(0.1737)	(0.1158)	(0.1895)	(0.1120)	(0.1329)
DT2	0.2772	0.2576	0.2386	0.2355	0.2986	0.2626
	(0.0884)	(0.1530)	(0.0795)	(0.1689)	(0.0936)	(0.1156)
DT3	0.2465	0.3521*	0.2722	0.2884	0.2532	0.5873*
	(0.1134)	(0.1096)	(0.1078)	(0.1797)	(0.1002)	(0.1088)
DT4	0.3604*	0.0764	0.3172	0.2607	0.3101	0.3307
	(0.1139)	(0.1365)	(0.1604)	(0.1250)	(0.2381)	(0.1126)
DT5	0.4754*	0.1847	0.4385*	0.4121*	0.3287	0.3482
	(0.1712)	(0.1100)	(0.1392)	(0.1340)	(0.1231)	(0.1579)
10 d fecundity	0.4243**	0.2366	0.5349*	0.2875	0.3357	0.6082*
	(0.1601)	(0.1571)	(0.2234)	(0.1693)	(0.2308)	(0.1985)

**Table 3:** Genetic correlations among life history traits for *Sitobion avenae* lines infected (above the diagonal) and uninfected (below the diagonal) with *Regiella insecticola* (genetic correlations were derived from variances calculated from combined data on wheat, oat and rye; *P < 0.05; **P < 0.01).

Traits	DT1	DT2	DT3	DT4	DT5	10 d fecundity
DT1	–	−0.0275	0.2028	0.0776	0.4979**	−0.2267
DT2	0.0488	–	0.0652	0.0941	0.5111**	−0.2856
DT3	0.1533	0.2753	–	0.1366	0.5493**	−0.3304
DT4	0.2414	0.1143	0.1243	–	0.6526**	−0.6194**
DT5	0.4987**	0.5448**	0.6764**	0.6536**	–	−0.6322**
10 d fecundity	−0.2273	−0.2261	−0.2993	−0.4662*	−0.4828*	–

**Table 4:** Comparisons of G-matrices for life-history traits of *Sitobion avenae* lines infected (U) and uninfected (NU) with *Regiella insecticola* (verdict, the best model in the Flury hierarchy that explained the structural differences between matrices; significant deviation from equality for the paired matrices indicated by *P*-values; CPC(1) and CPC(3), one and three of the six possible components shared in common; full CPC, all principal components shared in common).

G matrices	Test plant	Flury hierarchy		
		LRT	*P*-value	Verdict
U	Wheat	322.3	<0.001	CPC(3)
vs.	Oat	41.1	<0.001	CPC(1)
NU	Rye	34.2	0.035	Full CPC

**Table 5:** Selection differentials and gradients for life-history trait plasticities of *Sitobion avenae* lines infected and cured of *Regiella insecticola* on three host plants (DT1-DT4, the developmental time of 1^st^ to 4^th^ instar nymphs; DT5, the total developmental time of nymphs; *P < 0.05; **P < 0.01; ***P < 0.001).

Trait plasticities	Aphid clones cured of *R. insecticola*		Aphid clones infected with *R. insecticola*	
	Differential	Gradient	Differential	Gradient
DT1	0.2214***	−0.0289	−0.2085***	−0.2314*
DT2	−0.3031***	−0.0977	−0.2670***	−0.2073*
DT3	−0.2323***	−0.0549	0.1116	−0.1074
DT4	0.0026	0.1580	0.0163	0.1641
DT5	−0.1710**	0.0543	−0.2501***	0.1989
10 d fecundity	−0.3091***	−0.3434***	−0.3283***	−0.6303***

**Table 6:** Collection information and genotypes at four microsatellite loci for the aphid *Sitobion avenae* (U, *Regiella insecticola* infected aphid clones.

Clones	Collection locality (GPS coordinates)	Sm 10^a^	Sm 17^a^	Sm 12^a^	S4aΣ^a^
U2	Fuping Co. in Shaanxi (E 109^o^ 01′ 56″, N 34^o^ 46′ 46″)	166/166^b^	96/96	155/155	154/167
U3	Fuping Co. in Shaanxi (E 109^o^ 04′ 13″, N 34^o^ 45′ 21″)	150/160	94/100	*	169/169
U4	Chenggu Co. in Shaanxi (E 107^o^ 16′ 49″, N 33^o^ 07′ 50″)	155/166	96/102	147/157	165/167
U7	Huaying Co. in Shaanxi (E 110 ^o^ 05′ 08″; N 34 ^o^ 33′ 59″)	164/164	100/104	167/177	175/175
U9	Datong Co. in Qinghai (E101° 45′ 32″, N36° 47′ 59″)	155/164	100/105	137/137	164/164
U11	Datong Co. in Qinghai (E 101° 38′ 02″, N 36° 58′ 31″)	155/155	100/106	155/155	162/168
U14	Huzu Co. in Qinghai (E 101^o^ 57′ 30″, N 36 ^o^ 50′ 37″)	157/166	96/102	149/157	165/165

^*^Loss of alleles.

^a^Microsatellite loci.

^b^Allele sizes at each locus.
